# Prediction of Superheated Steam Temperature in Thermal Power Plants Based on the iTransformer Model

**DOI:** 10.3390/s26103078

**Published:** 2026-05-13

**Authors:** Yiyao Zhang, Feng Xie, Wei Shen, Xingyang Li, Chase Wu

**Affiliations:** 1 School of Computer Science and Technology, Zhejiang Sci-Tech University, Hangzhou 310018, China; 2024220603113@mails.zstu.edu.cn (Y.Z.); 2024220603088@mails.zstu.edu.cn (F.X.); 2023337621278@mails.zstu.edu.cn (X.L.); 2 Department of Data Science, New Jersey Institute of Technology, Newark, NJ 07102, USA

**Keywords:** superheated steam, temperature prediction, iTransformer, LSTM, time series, sensors

## Abstract

Accurate prediction of superheated steam temperature (SST) is critical for the safe and efficient operation of large-scale thermal power units, particularly under large load variations and high thermal inertia. This study proposes an iTransformer-based SST prediction framework (iTransformer-SST) to address limitations of conventional proportional–integral–derivative (PID) control and existing data-driven models in capturing multivariable coupling, time-delay effects, and physical consistency. Using the A-side subsystem of a 1000 MW thermal power unit, 19-dimensional process data were collected continuously over two months with a sampling interval of 2.4 s. After data preprocessing, time-lagged cross-correlation (TLCC) analysis combined with expert knowledge was employed for feature screening, resulting in ten highly relevant input variables. To enhance predictive robustness, the baseline iTransformer was augmented with a Local Temporal Convolution (LTC) module for local disturbance modeling and a physics-guided regularization term to enforce delayed monotonicity and smoothness constraints. In 240 min rolling forecasts of the final-stage superheater outlet temperature, the proposed model achieved a mean squared error (MSE) of 0.0887, a mean absolute error (MAE) of 0.2312, and a coefficient of determination ($R^{2}$) of 0.9650, significantly outperforming long short-term memory (LSTM), Informer, and the baseline iTransformer. The combined LTC and physics-guided design reduced MSE by 13.5%, demonstrating strong potential for feedforward-assisted SST control in industrial thermal power applications.

## 1. Introduction

Global electricity demand has continued to rise, while power systems must balance decarbonization targets with secure and flexible operation. Despite the rapid expansion of renewable generation, coal-fired power still contributes a substantial share of total electricity production (about one-third in recent IEA assessments) [1]. Under high-renewable and market-oriented dispatch conditions, coal-fired units are increasingly required to perform frequent and deep load-following.

In this operating context, superheated steam temperature (SST) exhibits stronger fluctuations, while strict safety limits must still be maintained to avoid thermal stress and efficiency loss. Accurate short-term SST forecasting is therefore a practical prerequisite for feedforward-assisted control in distributed control systems (DCSs). In industrial boiler systems, SST dynamics involve large inertia, multi-source disturbances, and variable time delays. These properties make conventional proportional–integral–derivative (PID)-based regulation difficult to tune and often insufficient during rapid load transitions. Existing data-driven studies have improved prediction performance, but their applicability to real-time industrial deployment remains limited by three core research gaps:

1. Gap 1: insufficient joint modeling of global coupling and local delay effects. Existing studies can be broadly categorized into three mainstream approaches:
  (a) Model Predictive Control (MPC) [2,3,4]: This approach relies heavily on accurate first-principles models and online state estimation. Abrupt variations in coal quality or load demand may lead to significant model–plant mismatch, thereby degrading control performance.
  (b) Temporal Convolutional Networks (TCN) [5]: TCNs are effective in capturing local temporal patterns; however, their limited receptive fields restrict their ability to model long-range dependencies. In addition, global interactions among process variables are often insufficiently represented.
  (c) Long Short-Term Memory Networks (LSTM) [4,6]: LSTM-based models are capable of modeling relatively long sequences, but they typically involve a large number of parameters and require substantial training time. Moreover, they are prone to gradient-related issues and error accumulation under non-stationary and noisy conditions, while their limited interpretability poses challenges for online monitoring and diagnosis.
2. Gap 2: limited physical consistency in sequence models. Many models optimize purely data-driven objectives and may generate predictions that are inconsistent with process monotonicity or lag behavior.
3. Gap 3: weak deployment orientation. Improvements in prediction accuracy are often not explicitly aligned with low-latency edge-side operation and DCS integration requirements.

To address these unmet needs, this paper proposes iTransformer-SST, an SST-oriented framework that explicitly links model design to industrial constraints. The framework builds on iTransformer but differs from prior applications in two key aspects: (i) a Local Temporal Convolution (LTC) path complements variable-wise attention with short-horizon local dynamics; and (ii) time-lag-aware physical priors are embedded in the loss function to regularize training toward physically plausible trajectories. Unlike generic physics-guided prediction methods, the proposed constraints are derived from identified SST lag characteristics and process monotonicity rather than only broad conservation heuristics.

From a deployment perspective, the proposed model is designed for edge-side inference under a 2.4 s sampling interval and direct interaction with feedforward and cascade PID loops in DCS environments [7]. This design follows the practical trend of moving latency-sensitive industrial intelligence from cloud to edge to reduce communication uncertainty and improve operational robustness [8,9].

The remainder of this paper is organized as follows. Section 2 reviews SST prediction and control methods in thermal power plants. Section 3 introduces the proposed architecture and loss design. Section 4 describes data collection, feature selection, and preprocessing. Section 5 presents the experimental setup and results, including model comparisons and ablation analysis. Section 6 concludes the paper and outlines future research directions.

## 2. Literature Review

Maintaining SST within a safe operating range is critical for ensuring the reliable and efficient operation of thermal power plants. Consequently, accurate SST prediction has long been a key challenge in automatic control systems.

In practical applications, cascade proportional-integral-derivative (PID) controllers remain the dominant approach for SST regulation in thermal power plants. However, due to the inherent characteristics of boiler systems, including large inertia, significant time delay, and multiple disturbances, determining optimal PID parameters remains challenging. As a result, conventional PID controllers often fail to meet control performance requirements, particularly under complex operating conditions, where issues such as overshoot and oscillation frequently occur. In extreme cases, rapid temperature increases may drive the cooling-water control signal to its upper limit while still failing to maintain SST within a safe range.

To address these limitations, substantial research has focused on enhancing PID-based control strategies with predictive and adaptive mechanisms. For instance, Yan et al. [2] proposed a hybrid control framework that combines cascade control with local Smith-predictor compensation, enabling more accurate process modeling and improved control performance. Similarly, Hu et al. [10] developed a multi-model weighted scheduling strategy for SST prediction and control, where multiple predictive models are integrated through a mechanism that dynamically assigns weights according to operating conditions, thereby enhancing robustness against disturbances.

However, these methods often fail to adequately capture the complex interactions among multivariate features. Given the highly nonlinear characteristics, strong spatiotemporal coupling, and delayed cross-variable effects in thermal power plant data, machine learning (ML) methods are particularly important because they can learn high-dimensional nonlinear mappings directly from operational data without requiring fully accurate first-principles models under every regime. In particular, deep neural network-based approaches have been increasingly adopted for SST prediction due to their ability to model long-range temporal dependencies and complex feature relationships.

Wang et al. [6] proposed a multi-mode switching strategy that incorporates an attention-based LSTM model. By introducing energy conservation as an equality constraint in the loss function, the method improves SST prediction accuracy on real-world thermal-power-plant datasets. Similarly, Huang et al. [4] developed a deep-learning-based predictive-control framework that integrates LSTM with MPC. This approach decouples predictive performance from mode-switching strategies and enables adaptive operation under varying conditions, achieving approximately a 10% reduction in overshoot while improving prediction accuracy.

Despite these advances, LSTM-based models exhibit inherent limitations when handling long time-series data [11,12]. In particular, their ability to capture long-term dependencies remains constrained, which has been identified as a key bottleneck in sequence modeling tasks [13]. Therefore, developing time-series models capable of effectively capturing and retaining complex long-range dependencies remains a critical challenge.

With the increasing adoption of the Transformer model [14] and its variants, attention-based architectures have become important tools for industrial time-series prediction. Their strength lies in modeling long-range dependencies and cross-variable interactions, but practical performance still depends on task-specific adaptation.

Recent iTransformer studies show this trend clearly. Liu et al. [15] introduced the variable-wise attention paradigm, and subsequent energy-domain work further adapted iTransformer for short-term electric load forecasting through feature-enhancement modules [16]. These studies confirm the efficiency of iTransformer-style backbones, but they focus mainly on generic forecasting benchmarks and do not explicitly address SST-specific delay structure, physical consistency, or control-oriented deployment requirements.

In parallel, physics-guided modeling has gained attention in thermal power applications. Beyond earlier SST studies that introduced physical constraints in hybrid predictors [6], recent work has explored physics-guided neural ODE forecasting for key thermal-power variables (e.g., main steam pressure), showing improved interpretability and control compatibility [17]. However, most existing physics-guided thermal-power methods are designed for variables other than SST or rely on model forms that are not tightly coupled with iTransformer-style multivariate representation learning.

Therefore, the key gap in related work is not whether iTransformer or physics-guided methods are effective in isolation, but how to couple them for SST forecasting under industrial constraints. Compared with prior studies, our framework explicitly combines: (i) iTransformer for global multivariable coupling, (ii) LTC for local delayed dynamics, and (iii) SST-specific physics-guided regularization for monotonicity and smoothness consistency. This synthesis directly links the reviewed methods to the proposed framework and clarifies the methodological advance over existing iTransformer and physics-guided thermal-power prediction studies.

The main contributions of this work are summarized as follows:

1. A real-world industrial dataset consisting of measurements from 19 sensor variables in a 1000 MW thermal power plant is utilized for model training and evaluation. The data are preprocessed through interpolation, smoothing, and time-lagged cross-correlation (TLCC) analysis, based on which the ten most relevant features are selected as model inputs.
2. An SST-oriented iTransformer framework is developed by integrating a Local Temporal Convolution (LTC) path with variable-wise attention, thereby improving joint modeling of multivariate coupling and local delayed dynamics.
3. A time-lag-aware physics-guided loss is introduced to enforce physically consistent prediction behavior. Distinct from generic physics-guided formulations, the regularization terms are directly derived from identified SST monotonicity and lag characteristics.
4. Extensive comparative and ablation experiments demonstrate that iTransformer-SST outperforms LSTM-, Informer-, and baseline iTransformer-based methods in both prediction accuracy and inference efficiency, while maintaining edge-deployment compatibility (measured average inference latency: 1.955 ms).

## 3. The Overall Framework of the Proposed Model

To address the aforementioned challenges, this paper proposes an iTransformer-based superheated steam temperature prediction model (iTransformer-SST), specifically designed to capture complex multivariate dependencies while maintaining high computational efficiency for real-time deployment.

This section first introduces the operational process of a coal-fired boiler unit and then presents the overall framework of the proposed method.

### 3.1. Thermal Power Unit Process

**Figure 1:**
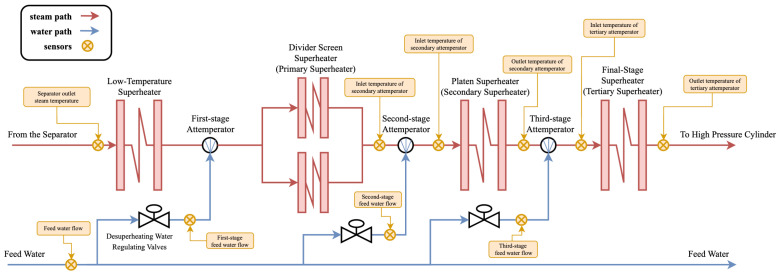
Schematic diagram of the reheat steam desuperheating system and the location of sensors.

For the 1000 MW thermal power unit considered in this study, the configuration of the steam superheating and desuperheating system is shown in Figure 1. The system consists of a low-temperature superheater, a divided screen superheater (primary superheater), a platen superheater (secondary superheater), and a final-stage superheater (tertiary superheater).

Steam from the separator flows sequentially through the primary, secondary, and tertiary superheaters, where it is progressively heated to the required degree of superheat before being delivered to the high-pressure cylinder. Throughout this process, steam temperature is regulated by adjusting the desuperheating-water valve to keep operation within safe limits.

The unit is structurally divided into two identical subsystems, denoted as side A and side B. Without loss of generality, only side A is considered in the subsequent analysis.

The superheater is a key boiler component that raises steam temperature from saturation to the required superheated level. It typically consists of multiple parallel tubes connected by inlet and outlet headers. These heating surfaces are located in the upper furnace and in the transition region between the radiative and convective zones, where they primarily absorb radiative heat from the furnace flame, supplemented by convective heat transfer from the flue gas.

The first-, second-, and third-stage attemperators regulate the temperatures of the divided screen superheater (primary), platen superheater (secondary), and final-stage superheater (tertiary), respectively, thereby preventing overheating at each stage. In addition, they mitigate temperature imbalances between parallel flow paths and maintain the main steam temperature at its rated value.

To avoid abrupt valve operations that may cause rapid fluctuations in superheater tube-wall temperature and accelerate material degradation, each attemperator must be controlled smoothly with sufficient anticipation [18].

The acquisition of key operating parameters (e.g., superheater temperature, pressure, and flow rate) relies on the distributed control system (DCS) [19] and a comprehensive sensor network. Measurements are collected at a sampling interval of 2.4 s using thermocouples, infrared and laser temperature sensors, pressure transmitters, and flow meters deployed throughout the system.

These data provide real-time monitoring of operating conditions, support safe and efficient operation, and enable early detection of potential faults.

The sensors are primarily deployed at the following locations:

1. Temperature measurement points: separator outlet; inlet and outlet of the first-, second-, and third-stage attemperators; and final-stage superheater outlet.
2. Pressure measurement points: inlet and outlet locations equipped with pressure transmitters.
3. Flow measurement points: a Venturi flowmeter for inlet steam and a vortex flowmeter for desuperheating water.

In total, measurements from 19 sensor variables are collected to construct the multivariate input for the SST prediction model.

**Figure 2:**
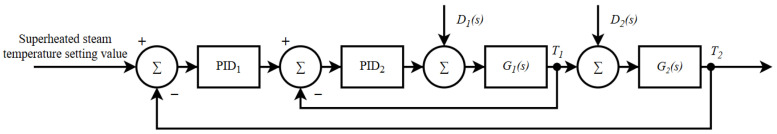
PID control system schematic diagram.

Currently, the SST of the unit is regulated using a cascade PID control strategy, as shown in Figure 2. The system input corresponds to the SST setpoint.

In this cascade control framework, $PID_{1}$ and $PID_{2}$ denote the primary and secondary controllers, respectively. $G_{1}\left(s\right)$ represents the transfer function of the mixing process between desuperheating water and high-temperature steam, while $G_{2}\left(s\right)$ describes the heat transfer dynamics of the superheater. $D_{1}\left(s\right)$ and $D_{2}\left(s\right)$ denote external disturbances acting on the steam side and the flue gas side, respectively [2].

Due to the strong coupling, time delay, and disturbance sensitivity of this cascade control system, accurate SST prediction is essential for improving control performance.

In practical thermal power plant applications, cascade PID control systems are typically deployed on edge-side industrial computing platforms located in close proximity to the distributed control system (DCS). These edge nodes are responsible for acquiring high-frequency measurements from temperature, pressure, and flow sensors, executing control-related computations, and interacting with actuators under strict real-time constraints.

However, due to the limited responsiveness of feedback-only PID control under significant thermal inertia and strong load variations, there is an increasing demand for predictive information that can be directly integrated into the edge-side control loop.

Motivated by this requirement, the proposed iTransformer-SST framework is designed for deployment on edge computing nodes as an auxiliary intelligence module for SST regulation. At the edge layer, incoming sensor data are first preprocessed through normalization and sliding-window construction, after which the trained model performs low-latency inference to generate short-term SST predictions.

These predictions are then provided to the cascade PID controller or MPC module as feedforward references, enabling earlier corrective actions and smoother temperature regulation. This integration enhances the responsiveness and stability of the control system under dynamic operating conditions.

Such an edge-centric deployment paradigm effectively decouples real-time prediction and control from cloud-based computation, thereby ensuring stable system operation even under network latency or interruptions [20].

Meanwhile, only aggregated statistics or model parameters are transmitted to the cloud for offline analysis and periodic model retraining, significantly reducing communication overhead while enhancing data security and privacy [21,22].

This architecture provides a practical foundation for deploying efficient deep learning models, such as iTransformer-SST, in real-world industrial environments.

### 3.2. Problem Description

The superheated steam system is characterized by large thermal inertia, significant time delays, and strong external disturbances, which make it challenging for the widely adopted cascade PID control strategy to maintain SST within the safe range of ±5 °C. In addition, PID parameter tuning heavily relies on operator experience, and manual intervention is often required under complex and varying operating conditions. When steam temperature rises rapidly, even fully opening the desuperheating-water control valve may fail to suppress the rise in time, which can lead to over-temperature conditions and threaten unit safety. Therefore, developing a more accurate and reliable SST prediction method is both practically significant and still urgent.

SST prediction can be formulated as a multivariate time-series forecasting task. Using a sliding-window scheme with a window length of *L*, the input sequence is constructed as $X=X_{i}^{t}|t=1,\ldots ,L;\;i=1,\ldots ,N$, where *N* denotes the number of input features, and $X_{i}^{t}$ represents the measurement of the *i*-th feature at time step *t*. Given these input sequences, the objective is to learn the nonlinear mapping between multivariate process variables and the target steam temperature and to predict future SST values over a forecasting horizon of length *T*.

### 3.3. Overview of the Proposed Framework

**Figure 3:**
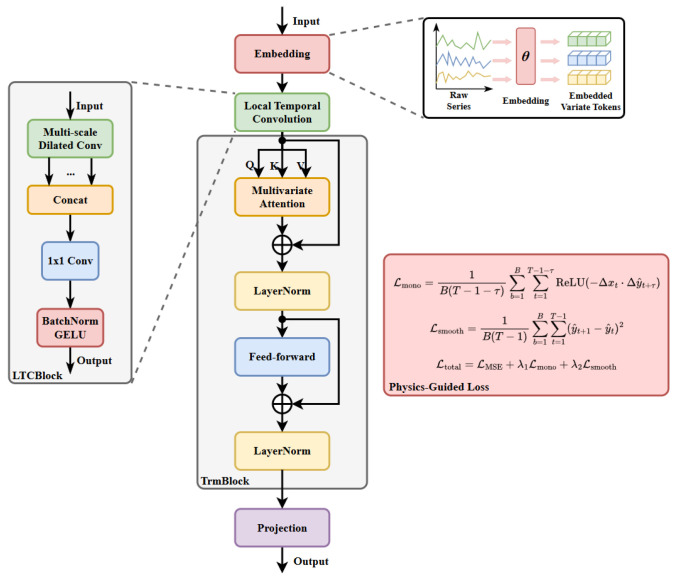
iTransformer-SST model framework.

To address the challenges described above, this paper proposes an integrated iTransformer-SST prediction framework that combines temporal convolution, the iTransformer backbone, and physics-guided constraints to improve prediction accuracy and operational efficiency. The overall architecture is shown in Figure 3. The main workflow of the proposed method is summarized as follows:

1. Data Collection and Preprocessing: A total of 19-dimensional operational data were collected from sensors deployed in a 1000 MW thermal power plant. The raw data were preprocessed using linear interpolation and smoothing techniques to handle missing values and noise, ensuring data quality for subsequent analysis.
2. Feature Selection and Analysis: TLCC analysis, combined with domain knowledge, was employed to evaluate the correlation between input variables and the target SST. Based on this analysis, the 10 most relevant features were selected, with time-lag effects explicitly considered to capture system dynamics.
3. Model Construction and Training: The proposed iTransformer-SST model was trained on the preprocessed data. It incorporates a LTC module to enhance local temporal feature extraction and introduces a physics-guided regularization term in the loss function to enforce consistency with steam temperature dynamics.
4. Prediction Execution: During inference, the model takes historical measurement data within the preceding 5 min as input and predicts the SST over the subsequent 1-minute horizon. The prediction process incorporates physical constraints to ensure consistency and reliability, while maintaining low latency for real-time deployment in thermal power plant control systems.

### 3.4. Design Rationale, Synergy Mechanism, and Methodological Justification

The proposed iTransformer-SST framework combines three components: (i) a Local Temporal Convolution (LTC) module, (ii) the iTransformer backbone, and (iii) physics-guided regularization. This design is motivated by the observation that SST prediction errors in thermal power systems originate from three distinct sources: short-term local disturbances, long-range cross-variable coupling, and physically inconsistent prediction trajectories.

First, the LTC module is introduced to capture local temporal dynamics (e.g., fast fluctuations caused by fuel-air disturbances and valve actions) that are difficult to model with variable-wise attention alone. Second, iTransformer is used as the global modeling backbone to learn multivariate dependency structures under strong coupling among process variables. Third, physics-guided regularization is added to constrain the output trajectory with monotonicity- and smoothness-related priors, improving physical plausibility and robustness under non-stationary operating conditions.

These components are complementary rather than redundant. LTC strengthens short-horizon pattern extraction, iTransformer models global inter-variable dependencies, and physics-guided constraints reduce physically implausible oscillations. Their combination forms a multi-level modeling strategy that jointly improves accuracy, generalization, and physical consistency.

We selected this combination after considering both predictive performance and deployment constraints in thermal power scenarios. Compared with heavier architectural alternatives, the proposed modifications introduce limited computational overhead while preserving real-time inference capability. Compared with purely data-driven objectives, physics-guided regularization provides additional inductive bias that is especially beneficial under disturbance-intensive operating regimes.

More importantly, these three modifications were selected over other potential enhancements based on task-specific suitability rather than model complexity. First, replacing the iTransformer backbone with larger temporal architectures (e.g., deeper full-sequence Transformers or stacked recurrent blocks) may increase representational capacity but typically incurs higher latency and weaker deployment efficiency for edge-side control support. Second, replacing LTC with deeper generic convolutional stacks may improve local fitting on some intervals but often provides limited gains relative to the additional parameters and tuning burden under fluctuating unit conditions. Third, replacing physics-guided regularization with purely statistical penalties can reduce short-term error, yet it does not explicitly constrain physically implausible trajectory behavior under disturbances. Therefore, the chosen design emphasizes a practical balance among accuracy, interpretability, robustness, and real-time feasibility, which aligns with the operational requirements of thermal power plants.

From a methodological perspective, the distinction from prior work is reflected at two levels. At the architectural level, we retain variable-wise attention as the global dependency modeling backbone and add a lightweight local temporal path to compensate for short-horizon disturbance sensitivity. At the objective-function level, we move beyond purely data-driven training by introducing delayed monotonicity and smoothness regularization derived from thermal process characteristics. This dual-level design differentiates the proposed framework from methods that modify only the network structure or only the loss formulation.

### 3.5. Local Temporal Convolution

The original iTransformer model takes an input sequence of the form $X\in R^{L\times D}$, where *L* denotes the sequence length and *D* represents the number of variables. The input is then transposed to $X^{T}\in R^{D\times L}$, such that each variable is treated as a token and the temporal dimension is regarded as the feature channel. This design facilitates the modeling of inter-variable dependencies and enables the attention mechanism to focus on relationships across different process variables.

While the self-attention mechanism in iTransformer is effective in capturing global dependencies, it may be less sensitive to localized temporal variations and short-term disturbances. However, in superheated steam systems, rapid fluctuations—such as changes in coal powder concentration and disturbances in desuperheating water injection—can have a significant impact on SST. These dynamics are often localized in time and require strong short-term feature extraction capability, which is not fully captured by attention-based models alone.

To address this limitation, the proposed iTransformer-SST introduces a LTC module in parallel with the self-attention mechanism. Specifically, the LTC module is designed to capture local temporal patterns through convolutional operations, thereby enhancing the model’s sensitivity to short-term variations. By integrating the LTC module with the global dependency modeling capability of self-attention, the proposed approach achieves improved responsiveness and robustness to local thermal disturbances under real-world operating conditions in thermal power plants.

The specific procedure of the LTC module is summarized as follows:

1. The input tensor $R^{B\times D\times L}$ is first processed by multi-scale dilated convolution, where multiple branches with different dilation rates are employed to capture temporal dependencies at varying receptive fields.
2. The feature maps generated by each convolution branch are concatenated along the channel dimension to aggregate multi-scale temporal information.
3. The concatenated features are projected back to the original dimensionality using a 1×1 pointwise convolution for feature fusion.
4. Finally, Batch Normalization (BatchNorm) and the Gaussian Error Linear Unit (GELU) activation function are applied to produce the output of the LTC module.

For reproducibility, the detailed LTC configuration used in this study is as follows: two parallel 1D convolution branches are adopted with dilation rates (1,2) and kernel size k=3. Each branch uses input and output channels equal to $d_{\mathrm{model}}=512$, with stride =1 and “same-length” padding p=d·(k−1)/2 for each dilation branch d∈{1,2}. The two branch outputs are concatenated into a tensor of size $B\times (2d_{\mathrm{model}})\times L$, and then projected by a 1×1 convolution from 1024 to 512 channels. BatchNorm and GELU are applied after feature fusion, followed by dropout (0.1) and residual addition with the embedded input to preserve training stability.

Compared with directly deepening the attention stack, the novelty of LTC in this work lies in introducing a lightweight, task-oriented local branch that is explicitly parallel to variable-wise global attention. This design targets short-horizon disturbance sensitivity in SST forecasting while preserving edge-side efficiency through limited additional parameters and simple fusion operations.

Its impact is quantified in the ablation study reported in Section 5. Relative to the baseline iTransformer, adding LTC reduces MSE from 0.1025 to 0.0899 (about 12.3%), reduces MAE from 0.2386 to 0.2329, and improves $R^{2}$ from 0.9595 to 0.9645. These gains show that LTC is not only an architectural addition but a functionally effective component for improving overall prediction performance under short-term disturbances.

### 3.6. Physics-Guided Loss

Traditional time-series prediction models typically rely on data-driven loss functions, such as mean squared error (MSE) or mean absolute error (MAE), for optimization. However, these approaches often fail to guarantee the physical consistency and interpretability of the model outputs.

To address this issue, this paper incorporates physical prior knowledge into the learning process by introducing a physics-guided loss function. Specifically, physical constraints are embedded into the loss function in the form of soft regularization terms, which guide the model toward physically consistent predictions and effectively reduce violations of fundamental physical principles.

Furthermore, previous studies have demonstrated the effectiveness of integrating physical constraints into the loss function; for example, Wang [6] showed that such constraints can significantly improve model reliability and physical consistency.

To enhance the physical consistency of the model predictions, a physics-guided loss function is constructed by augmenting the conventional data-driven loss with additional regularization terms derived from domain knowledge. Specifically, the overall loss function consists of a prediction error term and multiple physics-based constraints, which jointly guide the model toward physically plausible outputs.

The physical priors are quantified through a TLCC-based procedure. For each selected feature $x_{i}$, we compute its lagged correlation with SST and identify the dominant delay:

(1) $$\tau_{i}^{*}=\arg\max_{\tau \in [0,\tau_{\max}]}\left|\rho_{x_{i},y}\left(\tau \right)\right|,\;d_{i}=\mathrm{sign}\;\left(\rho_{x_{i},y}\left(\tau_{i}^{*}\right)\right)$$

where $\rho_{x_{i},y}\left(\tau \right)$ is the lagged correlation coefficient between feature $x_{i}$ and SST *y*, and $d_{i}\in \left\{+1,-1\right\}$ is the monotonicity direction indicator. The delay in physical units is converted to discrete time steps according to the sampling interval Δt=2.4 s:

(2) $$\tau_{i}=\max\;\left(1,\;\mathrm{round}\;\left(\frac{\tau_{i}^{*}}{\Delta t}\right)\right)$$

In practice, only variables with clear engineering causality and stable correlation signs are included in the monotonicity term, while weakly correlated variables are excluded to avoid introducing noisy physical constraints.

The regularization term consists of two physical constraints:

1. Delayed Monotonicity Constraint
  In the thermal power unit data used in this study, certain process variables (e.g., coal feed rate and air flow) exhibit a positive correlation with SST, with an inherent time delay (typically 1–5 min). That is, when other conditions remain unchanged, an increase in the input variable is expected to result in an increase in SST after a delay of τ. To capture this lagged relationship, a delayed monotonicity regularization term is formulated as follows:
  (3) $$L_{\mathrm{mono}}=\frac{1}{BK}\sum_{b=1}^{B}\sum_{i=1}^{K}\frac{1}{\tau_{i}}\sum_{t=T-\tau_{i}+1}^{T}\mathrm{ReLU}\left(-d_{i}\;\Delta x_{i,t}\;\Delta {\hat{y}}_{t+\tau_{i}}\right)$$
  Here, $x_{i,t}$ denotes the value of the *i*-th selected input feature at time *t*, and ${\hat{y}}_{t}$ denotes the predicted SST at time *t*. We define $\Delta x_{i,t}=x_{i,t+1}-x_{i,t}$ and $\Delta {\hat{y}}_{t+\tau_{i}}={\hat{y}}_{t+1+\tau_{i}}-{\hat{y}}_{t+\tau_{i}}$. *K* is the number of constrained features, $d_{i}$ is the sign indicator determined by Equation (1), and $\tau_{i}$ is the feature-specific delay in discrete steps determined by Equation (2). *B* is the batch size and *T* is the input length (i.e., seq_len). The ReLU operator penalizes violations of the expected delayed monotonic trend.
2. Smoothness Constraint
  Due to the thermal inertia of the superheated steam system, SST variations are inherently continuous and smooth. Therefore, a smoothness regularization term is introduced to penalize unrealistic rapid fluctuations in the predicted sequence, as defined in Equation (4):
  (4) $$L_{\mathrm{smooth}}=\frac{1}{B\hat{T}}\sum_{b=1}^{B}\sum_{t=T+1}^{T+\hat{T}}{\left({\hat{y}}_{t+1}-{\hat{y}}_{t}\right)}^{2}$$
  where $\hat{T}$ denotes the prediction horizon length. This term encourages temporal smoothness and improves the physical plausibility of the predicted SST trajectory.
  The final loss is defined as in Equation (5):
  (5) $$L_{\mathrm{total}}=L_{\mathrm{MSE}}+\lambda_{1}L_{\mathrm{mono}}+\lambda_{2}L_{\mathrm{smooth}}$$
  where $L_{\mathrm{MSE}}$ represents the standard prediction loss, and $\lambda_{1}$ and $\lambda_{2}$ are weighting coefficients that balance the contributions of the physics-based regularization terms.

The physics-guided terms are applied only during the training phase and are not included in the validation or test loss calculations. Experimental results demonstrate that the incorporation of physical regularization improves both the physical consistency and generalization performance of the model, particularly under operating conditions characterized by strong external disturbances and significant fluctuations.

The selection logic of $\lambda_{1}$ and $\lambda_{2}$ is as follows. We first perform Bayesian optimization in bounded ranges $\lambda_{1}\in \left[0,0.5\right]$ and $\lambda_{2}\in \left[0,0.3\right]$ together with architectural hyperparameters, using validation MSE as the primary objective. We then perform local manual refinement around the best region to ensure that the prediction trajectory remains physically smooth without over-constraining data fitting. The final setting ($\lambda_{1}=0.2$, $\lambda_{2}=0.1$) provides the best trade-off between numerical accuracy and physical plausibility in our validation experiments.

### 3.7. iTransformer

The iTransformer is an efficient Transformer-based architecture designed for time-series forecasting. Unlike standard Transformer models that treat time steps as tokens, iTransformer considers each variable (i.e., feature dimension) as a token and regards the temporal axis as the channel dimension. This design enhances the modeling of inter-variable dependencies in multivariate time series [15].

Compared with conventional Transformer architectures, iTransformer demonstrates superior performance in modeling high-dimensional and strongly coupled time series, making it particularly suitable for industrial applications such as sensor data analysis in thermal power plants. Its effectiveness stems from its ability to explicitly capture cross-variable interactions while preserving temporal information through channel-wise representations.

The overall iTransformer architecture consists of an embedding layer, a multi-head self-attention mechanism, a feed-forward network, and normalization layers with residual connections. The core components are detailed below.

#### 3.7.1. Embedding Layer

The input to the iTransformer is a multivariate time-series matrix $X\in R^{L\times D}$, where *L* denotes the number of time steps and *D* represents the number of variables. Unlike standard Transformer models, iTransformer first transposes the input to $X^{T}\in R^{D\times L}$, such that each variable is treated as a token and the temporal axis is regarded as the feature channel.

To preserve feature semantics and obtain a unified representation, a linear embedding layer is applied, as defined in Equation (6):

(6) $$E=XW_{E}+b_{E}$$

In practice, the input tensor is represented as $X\in R^{B\times L\times D}$, where *B* is the batch size. After transposition, the tensor is rearranged to $X\in R^{B\times D\times L}$ to align with the variable-as-token representation. The embedding operation projects each variable into a $d_{\mathrm{model}}$-dimensional space, resulting in an embedded representation $E\in R^{B\times D\times d_{\mathrm{model}}}$.

Here, $W_{E}$ and $b_{E}$ are learnable parameters. This embedding process enables the model to distinguish variables with different physical meanings (e.g., main steam pressure and generator power) while mapping them into a common feature space suitable for attention-based modeling.

In the proposed framework, a local temporal convolution module is further introduced after the embedding layer to enhance sensitivity to short-term disturbances (ranging from 10 s to 5 min), such as fluctuations in pulverized-coal concentration and transient variations in feedwater supply. This design improves the model’s capability in capturing local temporal patterns and enhances robustness under dynamic operating conditions.

#### 3.7.2. Multi-Head Self-Attention Mechanism

Given the high dimensionality, redundancy, and strong coupling of multivariate data in thermal power plants, complex inter-variable dependencies must be modeled effectively. To this end, a multi-head self-attention mechanism is used to capture relationships across multiple representation subspaces.

Specifically, the embedded input is linearly projected into multiple sets of query, key, and value vectors, corresponding to different attention heads. For each head, scaled dot-product attention is computed to model the interactions among variables in the transformed feature space. This process allows the model to learn diverse dependency patterns from different perspectives.

The outputs of all attention heads are then concatenated and projected to form the final representation. By aggregating information from multiple subspaces, the multi-head attention mechanism significantly enhances the model’s representation capability and its ability to capture complex dependencies in multivariate time-series data.

The core innovation of iTransformer lies in its inverted self-attention mechanism, which applies the query, key, and value computations along the variable dimension (i.e., each sensor or physical quantity), rather than across the temporal dimension as in conventional Transformer models. This variable-wise attention formulation enables more effective modeling of inter-variable dependencies in multivariate time series.

As a result, the model is particularly well suited for capturing the coupled effects of multiple input variables—such as main steam pressure, pulverized coal flow, and feedwater flow—on the target variable (SST) in thermal power units, thereby improving its ability to represent complex industrial process dynamics.

This design choice aligns well with the characteristics of industrial multivariate time series, where variable interactions play a more critical role than purely temporal dependencies.

The multi-head self-attention mechanism [23] operates as follows:

1. Linear Projection and Multi-Head Splitting
  Given the embedded feature representation *X*, the query, key, and value matrices are first obtained through three linear projections, as defined in Equation (7):
  (7) $$Q=XW_{Q},\;K=XW_{K},\;V=XW_{V}$$
  where $W_{Q},W_{K},W_{V}\in R^{d_{\mathrm{model}}\times d_{k}}$ are learnable projection matrices, $d_{\mathrm{model}}$ denotes the model dimension, and $d_{k}$ represents the dimensionality of the query and key vectors. Subsequently, *Q*, *K*, and *V* are split into *h* parallel subspaces according to the number of attention heads, resulting in ${Q_{i},K_{i},V_{i}}_{i=1}^{h}$. Each attention head operates on a distinct subspace, enabling the model to capture diverse patterns and dependencies from different representation perspectives.
2. Scaled Dot-Product Attention
  For each attention head, the attention scores are computed using the scaled dot-product operation, as defined in Equation (8):
  (8) $${\mathrm{score}}_{i}=\frac{Q_{i}K_{i}^{T}}{\sqrt{d_{k}}}$$
  where ${\mathrm{score}}_{i}$ denotes the attention score matrix for the *i*-th head, and $d_{k}=\frac{d_{\mathrm{model}}}{h}$ is the dimensionality of each attention head. The scaling factor $\sqrt{d_{k}}$ is introduced to prevent the dot-product values from becoming excessively large, which could otherwise lead to vanishing gradients after the softmax operation.
  The attention weights are then obtained by applying the softmax function to the attention scores:
  (9) $${\mathrm{attn}}_{i}=\mathrm{softmax}\left({\mathrm{score}}_{i}\right)$$
  where ${\mathrm{attn}}_{i}$ represents the attention weight matrix. In the standard formulation, it reflects the degree of interaction between tokens; in iTransformer, the tokens correspond to variables rather than time steps.
  Finally, the output of each attention head is computed as:
  (10) $${\mathrm{head}}_{i}={\mathrm{attn}}_{i}V_{i}$$
  where ${\mathrm{head}}_{i}$ denotes the output feature of the *i*-th attention head in its corresponding subspace.
3. Multi-Head Aggregation
  The outputs of the *h* attention heads are concatenated and projected back to the $d_{\mathrm{model}}$-dimensional space through a linear transformation, as defined in Equation (11):
  (11) $$\mathrm{MultiHead}\left(Q,K,V\right)=\mathrm{Concat}\left({\mathrm{head}}_{1},\ldots ,{\mathrm{head}}_{h}\right)W_{O}$$
  where $W_{O}\in R^{\left(h\cdot d_{v}\right)\times d_{\mathrm{model}}}$ is a learnable projection matrix, and $d_{v}$ denotes the dimensionality of the value vectors in each attention head.

Through parallel computation across multiple subspaces, the multi-head attention mechanism enables the model to effectively capture complex inter-variable dependencies in multivariate time-series data. In the context of thermal power systems, this design facilitates the modeling of coupled effects among multiple process variables, thereby improving the representation of dynamic system behavior.

Notably, in iTransformer, this mechanism operates along the variable dimension, further strengthening the modeling of cross-variable interactions.

#### 3.7.3. Feed-Forward Network

The feed-forward network (FFN) [24] is a key component of iTransformer that applies nonlinear transformations to the outputs of the attention mechanism. While the attention module captures global dependencies among variables, the FFN enhances the model’s representation capability through nonlinear feature transformations.

Following the standard Transformer architecture, the FFN consists of two linear transformations with an activation function in between, as defined in Equation (12):

(12) $$\mathrm{FFN}\left(x\right)=W_{2}\mathrm{GELU}\left(W_{1}x+b_{1}\right)+b_{2}$$

where $x\in R^{B\times L\times d_{\mathrm{model}}}$ denotes the FFN input (i.e., the output of the attention module), $W_{1}\in R^{d_{\mathrm{model}}\times 4d_{\mathrm{model}}}$ and $W_{2}\in R^{4d_{\mathrm{model}}\times d_{\mathrm{model}}}$ are learnable weight matrices, and $b_{1}$ and $b_{2}$ are bias terms. The Gaussian Error Linear Unit (GELU) is used as the activation function because its smooth nonlinearity is well suited to modeling continuous-valued time-series signals.

The FFN expands the feature dimension to $4d_{\mathrm{model}}$ in the hidden layer, enabling the model to capture more complex patterns in the data. Notably, the FFN operates independently on each position (i.e., each token) without introducing additional interactions across variables or time steps. This design improves computational efficiency while refining the representations learned by the attention mechanism.

#### 3.7.4. Normalization and Residual Connections

To stabilize deep-network training and improve convergence, iTransformer employs normalization and residual connections within each sublayer. These techniques alleviate vanishing gradients and facilitate efficient information flow across layers. The implementation is as follows:

1. Residual Connections
  Residual connections are introduced to link the input and output of each sublayer, including the multi-head self-attention and feed-forward network (FFN) modules.
  The attention sublayer is defined as:
  (13) $$x^{\prime }=x+\mathrm{MultiHead}\left(Q,K,V\right)$$
  The FFN sublayer is defined as:
  (14) $$x^{\prime \prime }=x^{\prime }+\mathrm{FFN}\left(x^{\prime }\right)$$
  where *x* denotes the input to the sublayer. The residual connections enable direct information propagation and improve gradient flow, thereby facilitating the training of deeper architectures.
2. Layer Normalization
  To stabilize feature scaling and improve optimization dynamics, iTransformer applies layer normalization after each residual connection, as defined in Equation (15):
  (15) $$\mathrm{LayerNorm}\left(x\right)=\frac{\gamma (x-\mu )}{\sqrt{\sigma^{2}+\varepsilon }}+\beta$$
  where μ and $\sigma^{2}$ denote the mean and variance of the features computed along the $d_{\mathrm{model}}$ dimension, respectively:
  (16) $$\mu =\frac{1}{d_{\mathrm{model}}}\sum_{i=1}^{d_{\mathrm{model}}}x_{i},\;\sigma^{2}=\frac{1}{d_{\mathrm{model}}}\sum_{i=1}^{d_{\mathrm{model}}}{\left(x_{i}-\mu \right)}^{2}$$
  Here, γ and β are learnable scaling and shifting parameters, and ε is a small constant added for numerical stability.
  Compared with batch normalization, layer normalization computes statistics independently for each sample rather than across a batch, making it more suitable for time-series data with non-stationary distributions.

## 4. Data Processing

### 4.1. Experimental Dataset

The industrial time-series dataset used in this study was collected from the distributed control system (DCS) of a 1000 MW ultra-supercritical coal-fired power generation unit in Zhejiang Province, China. The dataset covers the period from 15 February to 18 April 2025.

Although this study uses one unit for model development, the selected unit type is representative of mainstream large-capacity coal-fired units operating under deep load regulation in China. In addition, the collected period covers sustained operation under varying load and disturbance conditions, which supports evaluation under non-stationary industrial scenarios. Nevertheless, we acknowledge that cross-unit generalization cannot be fully established from a single-unit dataset and should be further verified through multi-unit data in future work.

**Figure 4:**
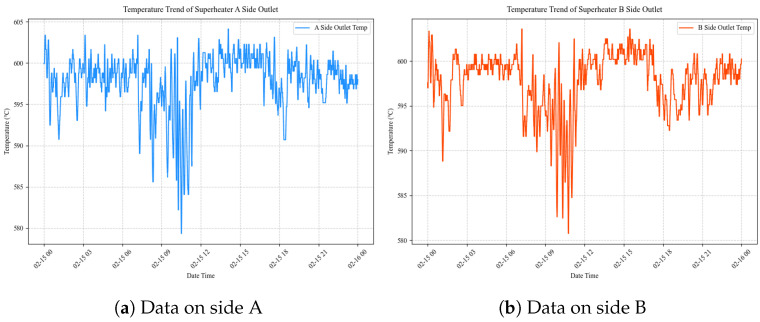
The Outlet Temperature Data of the Final-Stage Superheater on 15 February.

For model development and evaluation, the dataset was divided into training, validation, and test sets with a 7:1:2 split. The outlet temperature of the final-stage superheater on 15 February is shown in Figure 4.

The data were continuously recorded at a fixed sampling interval of 2.4 s, resulting in approximately 36,000 samples per day. In total, the dataset contains 2,268,000 records (63 days × 36,000/day). The DCS monitors unit operation by collecting 38 key process variables from both the A-side and B-side subsystems, covering boiler temperature regulation, fuel supply, airflow control, and energy output.

Since the A-side and B-side subsystems are structurally identical and exhibit consistent behavior in our analysis, this study focuses on the 19 process variables from side A in the subsequent experiments. The detailed variable list and their engineering significance are provided in Section 4.2.

The prediction targets are key parameters that reflect the safe operating status of the boiler superheater:

- Outlet temperature of the final superheater on side A (°C)
- Outlet temperature of the final superheater on side B (°C)

The dataset spans a wide range of operating conditions over the two-month period and exhibits typical characteristics of industrial time-series data, including high sampling frequency, strong multivariate coupling, and pronounced temporal dynamics with inherent delays. These properties make it a realistic and valuable benchmark for evaluating iTransformer-based prediction of key parameters in complex thermal power systems.

### 4.2. Feature Selection

#### 4.2.1. Feature Selection Objectives and Methods

Feature selection is performed to enhance prediction accuracy and improve model interpretability by removing redundant and irrelevant variables while preserving key physical information. In this study, a multi-stage hybrid strategy combining domain knowledge and data-driven analysis is used to determine the input features.

1. Domain Knowledge Guidance
  Based on expert knowledge from power plant operation, candidate variables that are physically relevant to superheater temperature control are first identified. This step ensures that the selected features are closely associated with the underlying thermodynamic processes and reduces the inclusion of spurious or non-informative variables.
  This knowledge-driven pre-selection step provides a physically meaningful feature space for subsequent data-driven refinement.
2. Statistical Correlation Analysis
  To quantitatively evaluate the relevance between candidate features and the target variable (i.e., the outlet temperature of the final superheater), multiple correlation metrics are employed to capture different types of dependencies, including linear and monotonic relationships.
  (a) Pearson Correlation Coefficient (Linear Dependency)
    The Pearson correlation coefficient is used to measure linear relationships between variables, as defined in Equation (17):
    (17) $$r=\frac{\mathrm{cov}(X,Y)}{\sigma_{X}\sigma_{Y}}=\frac{\sum_{i=1}^{n}\left(X_{i}-\bar{X}\right)\left(Y_{i}-\bar{Y}\right)}{\sqrt{\sum_{i=1}^{n}{\left(X_{i}-\bar{X}\right)}^{2}}\;\sqrt{\sum_{i=1}^{n}{\left(Y_{i}-\bar{Y}\right)}^{2}}}$$
    where $X_{i}$ and $Y_{i}$ denote the *i*-th observations of the feature and target variables, respectively, and $\bar{X}$ and $\bar{Y}$ are their sample means.
  (b) Spearman Rank Correlation Coefficient (Monotonic Dependency)
    The Spearman rank correlation coefficient is used to measure monotonic relationships, as defined in Equation (18):
    (18) $$r_{s}=1-\frac{6\sum_{i=1}^{n}d_{i}^{2}}{n(n^{2}-1)}$$
    where $R(X_{i})$ and $R(Y_{i})$ denote the ranks of $X_{i}$ and $Y_{i}$, respectively, and $d_{i}=R\left(X_{i}\right)-R\left(Y_{i}\right)$ is the rank difference for each observation pair. *n* is the number of samples.
3. Redundancy Control and Complementarity Verification
  To reduce multicollinearity, highly redundant candidate variables are pruned by jointly considering pairwise correlation, lag-pattern similarity, and engineering substitutability. When two variables exhibit highly similar statistical behavior and physical meaning, only the one with stronger relevance to the target and clearer physical interpretation is retained.
  Complementarity among retained features is then verified from two perspectives: (i) heterogeneous physical roles across the combustion–heat-transfer–desuperheating chain, and (ii) differentiated lag-time characteristics identified by TLCC. This procedure helps ensure that the final feature set contains non-redundant and mutually informative variables rather than repeated proxies of the same process factor.
4. Time-Lagged Cross-Correlation Analysis
  To capture the inherent time-delay characteristics of thermal power systems, time-lagged cross-correlation analysis is performed using the cross-correlation function (CCF). This method quantifies the dynamic response delay of each feature relative to the target variable.
  The cross-correlation function is defined as follows:
  (19) $$\mathrm{CCF}\left(\tau \right)=\frac{1}{\sigma_{X}\sigma_{Y}}\sum_{t=1}^{N-\tau }\left(X_{t}-\bar{X}\right)\left(Y_{t+\tau }-\bar{Y}\right)$$
  where τ denotes the lag time, and $\sigma_{X}$ and $\sigma_{Y}$ are the standard deviations of the feature and target variables, respectively.
  The optimal lag time $\tau^{*}$ is determined by maximizing the absolute value of the cross-correlation function:
  (20) $$\tau^{*}=\arg\max_{\tau }\left|\mathrm{CCF}(\tau )\right|$$
  This approach identifies delayed dependencies between variables, which are critical for modeling the dynamic behavior of superheated steam temperature.

Finally, the optimal feature set is determined by jointly considering statistical relevance and physical interpretability based on engineering knowledge.

#### 4.2.2. Feature Analysis Results and Feature Selection

Based on the aforementioned domain knowledge and correlation analyses, a subset of 10 representative features is selected from the original candidate variables. These features exhibit strong statistical relevance to the target variable as well as clear physical interpretability in the context of superheater temperature control.

The selected feature set is denoted as $X=(f_{1},f_{2},\ldots ,f_{10})$, where $f_{i}$ represents the *i*-th feature. The detailed list of selected features and their corresponding physical meanings are provided in Table 1.

The final selection is determined by jointly considering correlation strength, time-lag characteristics, engineering relevance, and redundancy control. This compact feature set reduces input redundancy while preserving key dynamic and physical characteristics, thereby providing an effective input representation for subsequent model training.

The correlation results for the selected features are summarized in Table 2 and Table 3, corresponding to the A-side and B-side subsystems, respectively.

For the B-side subsystem, the same analysis procedure is applied, and the results are presented in Table 3, where $f_{1}$ to $f_{4}$ correspond to the steam temperature variables on side B.

Notably, the correlation patterns observed for the A-side and B-side subsystems are highly consistent. This consistency further validates the reliability of the proposed feature selection strategy and confirms the structural symmetry between the two subsystems. This also indicates that the selected features capture the intrinsic physical relationships governing the superheater temperature dynamics.

As shown in Table 2 and Table 3, variations in fuel and air flow exhibit delayed effects on superheater temperature, with typical lag times exceeding 4 min. This observation is consistent with the thermal inertia characteristics of the boiler system.

More importantly, the selected variables exhibit differentiated lag distributions (approximately 0.88–4.60 min across both subsystems), indicating that they contribute complementary dynamic information at different temporal scales rather than duplicating a single response pattern.

In addition, oxygen content is negatively correlated with temperature. This behavior can be explained by combustion thermodynamics: an increase in oxygen content, often associated with higher total air flow, leads to excess-air conditions. Under such conditions, furnace flame temperature tends to decrease, thereby reducing radiative heat transfer and lowering flue-gas enthalpy in the convection superheater region.

The agreement between statistical observations and physical principles further demonstrates the effectiveness of the proposed feature-selection method.

### 4.3. Data Preprocessing

#### 4.3.1. Outlier Detection and Treatment

To ensure data quality, outliers are identified and processed using a dynamic thresholding method based on the Z-score [25], as defined in Equation (21):

(21) $$Z=\frac{|x_{i}-\mu |}{\sigma }$$

where μ and σ denote the mean and standard deviation of the feature, respectively. A threshold of |Z|>3 is used to detect outliers.

The outlier handling procedure consists of the following steps:

1. Dual-Channel Detection
  Outliers are independently detected in both the input features *X* and the target variable *Y*, as defined in Equation (22):
  (22) $${𝒪}_{X}=\left\{x_{i}|Z_{X}\left(i\right)>3\right\},\;{𝒪}_{Y}=\left\{y_{i}|Z_{Y}\left(i\right)>3\right\}$$
2. Joint Processing
  The final outlier set is obtained by taking the union of the detected outliers:
  (23) $$𝒪={𝒪}_{X}\cup {𝒪}_{Y}$$
3. Linear Interpolation
  The detected outliers are replaced using time-domain linear interpolation, as defined in Equation (24):
  (24) $$x_{t}^{\prime }=\frac{\left(t-t_{k-1}\right)x_{k}+\left(t_{k}-t\right)x_{k-1}}{t_{k}-t_{k-1}}$$
  where $t_{k-1}$ and $t_{k}$ denote adjacent valid (non-outlier) time points.

This preprocessing step improves data consistency and reduces the impact of abnormal fluctuations on subsequent model training.

#### 4.3.2. Robust Normalization

To mitigate the impact of scale differences across variables, robust normalization is applied to the dataset. This method is based on the median and interquartile range (IQR), and is defined in Equation (25):

(25) $$x^{\prime }=\frac{x-\mathrm{Med}(X)}{\mathrm{IQR}(X)}$$

where Med(X) denotes the median of the feature, and $\mathrm{IQR}\left(X\right)=Q_{3}\left(X\right)-Q_{1}\left(X\right)$ is the interquartile range.

Compared with standard normalization methods based on the mean and standard deviation, this approach is less sensitive to extreme values because both the median and IQR are robust statistics. As a result, it provides more stable feature scaling for industrial time-series data with potential outliers and non-Gaussian distributions. This is particularly important for thermal-power-plant data, where abrupt disturbances and sensor noise may introduce extreme values.

#### 4.3.3. Sliding Window Construction

To transform the continuous time-series data into a supervised learning format, a sliding-window strategy is used to construct input–output pairs for model training.

Specifically, given a multivariate time series ${\left\{X_{t}\right\}}_{t=1}^{T_{\mathrm{total}}}$ with *N* variables, an input sequence of length *L* is defined as:

(26) $$X^{\left(t\right)}=\left\{X_{t-L+1},X_{t-L+2},\ldots ,X_{t}\right\}\in R^{L\times N}$$

where $X_{t}\in R^{N}$ denotes the feature vector at time step *t*, and *L* is the input sequence length.

The corresponding prediction target is defined over a future horizon of length $\hat{T}$:

(27) $$Y^{\left(t\right)}=\left\{y_{t+1},y_{t+2},\ldots ,y_{t+\hat{T}}\right\}$$

where $y_{t}$ represents the target variable (i.e., superheated steam temperature) at time step *t*.

By sliding the window along the time axis with a fixed stride (typically one time step), a large number of input–output pairs are generated for model training. This approach enables the model to learn temporal dependencies and dynamic patterns in the data, which are essential for accurate prediction of superheated steam temperature under varying operating conditions.

## 5. Experiments and Performance Evaluation

### 5.1. Experimental Environment

All experiments were conducted on a workstation equipped with an NVIDIA GeForce RTX 4070 Ti SUPER GPU (NVIDIA Corporation, Santa Clara, CA, USA) and an Intel(R) Core(TM) i7-14700F CPU (Intel Corporation, Santa Clara, CA, USA). The software environment used Python 3.9.19 (Python Software Foundation, Wilmington, DE, USA) and PyTorch 2.4.1 (PyTorch Foundation, Linux Foundation, San Francisco, CA, USA).

### 5.2. Deployment Feasibility Quantification

To quantitatively assess practical deployability using the existing experimental setting, we report key operational indicators derived directly from the current data acquisition and inference configuration.

Given a sensor sampling interval of 2.4 s, the online prediction module has an update budget of 2400 ms per control cycle. In our deployment benchmark (excluding the first five warm-up inferences), the proposed iTransformer-SST achieves an average inference latency of 1.955 ms. Under this setting, the average inference time occupies about 0.08% of the per-cycle budget (1.955/2400), leaving more than 99.9% of the cycle for data communication, decision logic, and control actuation.

In addition, the model uses a 5 min historical input window (125 time steps) to produce 1 min-ahead predictions, which is consistent with the observed process delay range (up to approximately 4.6 min) and therefore compatible with feedforward-assisted control requirements. From an online-computing perspective, this configuration offers a favorable trade-off between temporal context coverage and runtime overhead, supporting stable real-time operation in latency-sensitive thermal power scenarios.

Overall, these quantitative indicators indicate that the proposed framework satisfies the basic timing constraints of edge-side deployment for SST prediction while maintaining sufficient prediction horizon for practical control support.

From an engineering evaluation perspective, deployment readiness is judged by three indicators: (i) per-step inference latency relative to the 2.4 s control-cycle budget, (ii) runtime memory footprint, and (iii) computational overhead under continuous rolling prediction. In this work, we provide explicit latency-budget analysis based on the deployed sampling configuration. A more comprehensive cross-model comparison of memory and long-horizon runtime overhead will be included in future multi-scenario evaluations.

Using the test-set deployment benchmark (after excluding the first five warm-up inferences), the measured online indicators are: average latency of 1.955 ms, 95th-percentile latency of 2.387 ms, maximum latency of 3.090 ms, average system memory of 930.951 MB (max 931.102 MB), average GPU memory of 37.720 MB (max 37.720 MB), average throughput of 12,880.578 steps/s, and rolling throughput at the end of the run of 12,570.721 steps/s.

Under the 2400 ms control-cycle budget, the average latency occupies only about 0.08% (1.955/2400), and even the observed maximum latency occupies about 0.13% (3.090/2400), leaving sufficient timing margin for communication and control execution. In addition, the low and stable GPU-memory footprint indicates that the proposed model remains lightweight from an edge-deployment perspective.

### 5.3. Interpretability Analysis

To improve model transparency, we analyze interpretability from three complementary perspectives: (i) physically grounded feature semantics, (ii) lag consistency between data analysis and model design, and (iii) component-level contribution attribution.

First, the input features are not selected solely by statistical relevance. As described in Section 4.2, feature screening jointly considers domain knowledge, redundancy control, and complementarity verification. Each retained variable therefore has a clear engineering role in the combustion–heat-transfer–desuperheating chain, providing a physically interpretable basis for model inputs rather than opaque high-dimensional proxies.

Second, temporal interpretability is strengthened through TLCC-based lag analysis and physics-guided regularization. The identified feature-specific lag characteristics ($\tau_{i}$) are explicitly incorporated into the delayed monotonicity constraint (Equations (1)–(3)), while thermal inertia is reflected in the smoothness term (Equation (4)). This design links the model objective to known process dynamics and reduces physically implausible trajectory behavior.

Third, we use ablation-based attribution to quantify the contribution of each interpretable module. As discussed in the ablation analysis in Section 5, introducing LTC improves short-horizon disturbance modeling, and adding physics-guided loss provides additional gains in both accuracy and physical consistency. This module-wise evidence makes the performance improvement traceable to specific, interpretable design choices rather than a black-box architectural expansion.

**Figure 5:**
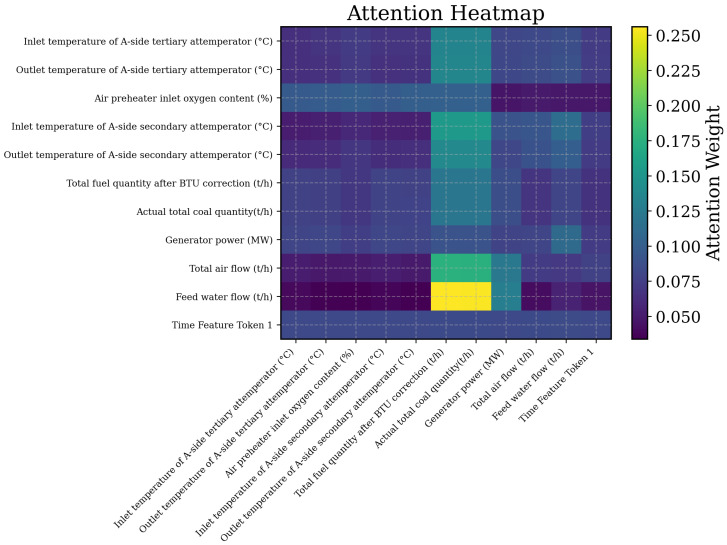
Variable-wise attention heatmap of iTransformer-SST. The matrix is averaged over test samples (and attention heads/layers) to show representative inter-variable dependency patterns learned by the model in the test set. Brighter colors indicate larger attention weights.

To provide explicit model-level evidence, we further visualize the averaged variable-wise attention matrix of iTransformer-SST (Figure 5). The heatmap reveals structured rather than uniform attention allocation. Relatively high weights are observed around fuel-related variables (total fuel quantity after BTU correction and actual total coal quantity) and feedwater flow, indicating that the model focuses on core process factors in the combustion–heat-transfer–desuperheating chain. Interactions between attemperator temperature variables and fuel/flow variables also show elevated attention weights, consistent with established process couplings in SST regulation. These observations provide direct interpretability evidence that complements the physics-guided objective and ablation analysis.

Overall, the proposed framework does not rely on post-hoc explanation alone; instead, interpretability is embedded into input construction, objective formulation, and component-level verification.

### 5.4. Evaluation Metrics

During training, the proposed physics-guided loss function is used to incorporate physical constraints into model optimization (see Equations (3)–(5)).

For performance evaluation, three widely used metrics are adopted: mean squared error (MSE), mean absolute error (MAE), and the coefficient of determination ($R^{2}$). These metrics provide complementary perspectives on prediction accuracy and goodness of fit.

The corresponding definitions are given as follows:

(28) $$\mathrm{MSE}=\frac{1}{n}\sum_{i=1}^{n}{\left(y_{i}-{\hat{y}}_{i}\right)}^{2},$$

(29) $$\mathrm{MAE}=\frac{1}{n}\sum_{i=1}^{n}\left|y_{i}-{\hat{y}}_{i}\right|,$$

(30) $$R^{2}=1-\frac{\sum_{i=1}^{n}{\left(y_{i}-{\hat{y}}_{i}\right)}^{2}}{\sum_{i=1}^{n}{\left(y_{i}-\bar{y}\right)}^{2}}$$

where *n* denotes the number of evaluation samples, $y_{i}$ is the ground-truth value, ${\hat{y}}_{i}$ is the predicted value, and $\bar{y}$ is the sample mean.

MSE emphasizes larger errors due to the squared term, MAE provides a direct measure of average absolute deviation, and $R^{2}$ reflects the proportion of variance explained by the model.

### 5.5. Effects of Hyperparameters

To determine an appropriate set of training hyperparameters (e.g., number of epochs, learning rate, and batch size), a series of comparative experiments is conducted.

For the look-back window length seq_len, the correlation analysis results in Table 2 and Table 3 indicate that the maximum time lag among the selected input features is approximately 4.6 min. Accordingly, seq_len is set to 5 min (i.e., 125 time steps), ensuring that the relevant historical dependencies affecting the target variable are fully captured.

#### 5.5.1. Epochs

The number of training epochs determines how many passes the model makes over the dataset and has a significant impact on convergence behavior. To investigate its effect, four settings {5, 10, 20, 30} are evaluated, with early stopping applied (patience = 10).

The experimental results are summarized in Table 4. When the maximum number of epochs is set to 30, early stopping is triggered, indicating that the model converges before reaching the upper limit. The best performance is achieved at 20 epochs, yielding the highest $R^{2}$ and the lowest MSE.

When the number of epochs is below 20, the model fails to adequately learn the underlying data patterns, resulting in underfitting. In contrast, increasing the number of epochs beyond 20 leads to performance degradation, suggesting the onset of overfitting.

#### 5.5.2. Learning Rate

The learning rate controls the step size of parameter updates during training and plays a critical role in model convergence. To evaluate its impact, multiple learning rates are tested, and the results are summarized in Table 5.

Among the tested settings, a learning rate of $10^{-4}$ achieves the best overall performance, yielding the highest $R^{2}$ along with relatively low MSE and MAE. This indicates an effective balance between convergence speed and prediction accuracy.

When smaller learning rates are used, the model converges more slowly and may fail to reach an optimal solution within the given training epochs. In contrast, larger learning rates lead to unstable training dynamics and performance degradation, likely due to overshooting during parameter updates.

This result highlights the importance of selecting an appropriate learning rate to ensure stable optimization and robust model performance.

#### 5.5.3. All Hyperparameters

All hyperparameter configurations are summarized in Table 6.

Considering the scale of the dataset and the strong coupling among variables, sufficient attention capacity is required to capture complex dependencies. Therefore, the number of attention heads is set to 8, providing a balance between representational capability and computational efficiency. The model dimension ($d_{\mathrm{model}}$) is set to 512, projecting the input features into a higher-dimensional latent space and enabling effective modeling of complex feature interactions.

The feed-forward dimension follows the standard Transformer configuration, with $d_{\mathrm{ff}}=4\times d_{\mathrm{model}}$, ensuring adequate capacity for nonlinear transformations. The number of encoder layers ($e_{\mathrm{layers}}$) is set to 2 to learn hierarchical feature representations while maintaining a reasonable computational cost.

To incorporate domain knowledge, two physics-guided regularization terms are introduced, with weights $\lambda_{1}$ and $\lambda_{2}$, corresponding to the monotonicity and smoothness constraints, respectively. These hyperparameters, together with the learning rate and architectural settings, are first optimized via Bayesian optimization and then further refined through empirical tuning.

The dropout rate is fixed at 0.1 to improve generalization without significantly reducing model capacity. Experimental results indicate that increasing the dropout rate to 0.3 leads to noticeable performance degradation, suggesting that excessive regularization may hinder the model’s ability to capture higher-order temporal dependencies.

Overall, the selected hyperparameter configuration achieves a balance between model complexity, training stability, and generalization performance.

### 5.6. Experiments on the Power Unit Dataset

#### 5.6.1. Comparison of Different Models

Although the proposed model performs one-step-ahead forecasting (1 min) based on a 5 min historical window, evaluation is conducted in a rolling manner to better reflect continuous predictive performance in practical scenarios. Specifically, a continuous 240 min (4 h) segment is selected from the test set, and 1 min-ahead predictions are generated sequentially at each time step. The evaluation metrics are then averaged over the entire 4 h horizon, ensuring statistical robustness and enabling a fair comparison across models.

To standardize comparison conditions, all baseline models and the proposed model use the same dataset split, identical input/output horizon definitions, the same preprocessing pipeline, and the same evaluation metrics. Hyperparameters for each model are selected on the validation set according to the same train/validation protocol, and the final test comparison is conducted only after validation-based model selection.

To further assess the practical effectiveness of the proposed method, several widely used time-series forecasting models are selected as benchmarks for comparison. All models are evaluated on the same 240 min (4 h) test segment under identical experimental conditions to ensure consistency. Mean squared error (MSE), mean absolute error (MAE), and the coefficient of determination ($R^{2}$) are adopted as evaluation metrics. The comparative results are summarized in Table 7.

**Figure 6:**
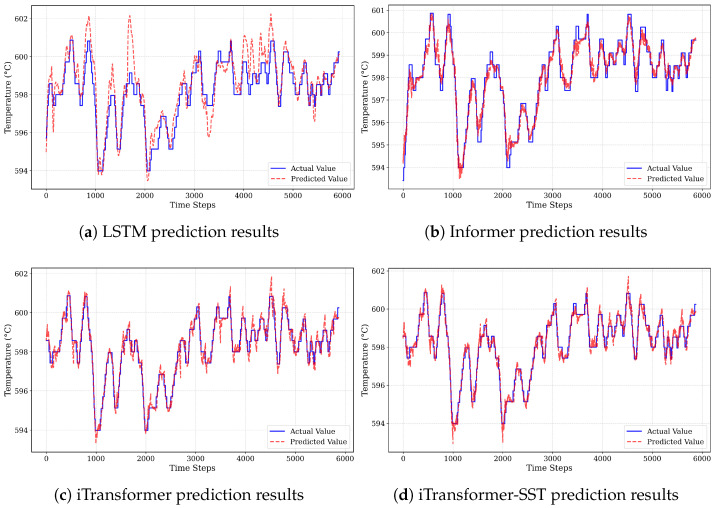
Prediction results.

The continuous prediction results of all models are shown in Figure 6.

The results indicate that LSTM performs poorly in SST forecasting under complex multivariate conditions. Its MSE and MAE are significantly higher, while its $R^{2}$ is notably lower than those of the other models, suggesting a limited ability to capture the highly nonlinear and strongly coupled dynamics inherent in coal-fired power plant processes.

In contrast, Informer demonstrates improved fitting performance, achieving an $R^{2}$ of 0.9461 along with reduced MSE and MAE. This improvement can be attributed to its capability to model temporal dependencies and mitigate long-range dependency issues. However, its predictions still exhibit noticeable fluctuations, and its performance remains constrained under complex and non-stationary operating conditions.

By inverting the attention dimension and tailoring the architecture for time-series forecasting, iTransformer further enhances predictive performance. It achieves an $R^{2}$ of 0.9595 with lower MSE and MAE, demonstrating a stronger ability to model high-dimensional multivariate features and capture inter-variable dependencies.

Notably, the proposed iTransformer-SST achieves the best overall performance across all evaluation metrics, with further reductions in MSE and MAE and an increased $R^{2}$ of 0.9650. As shown in Figure 6d, the prediction error remains within ±2 °C throughout the evaluated period, even under extreme load fluctuations, indicating robust behavior relative to the baseline models. This improvement is consistent with the contributions of the Local Temporal Convolution (LTC) path and the physics-guided loss, which are designed to better capture coupling relationships, time-lag effects, and non-stationary behavior in multivariate process variables. Although the proposed method introduces a slight increase in computational cost, the accuracy gain has clear practical value for high-reliability applications such as steam-temperature control in thermal power plants.

Overall, the results demonstrate that the proposed iTransformer-SST provides a more accurate and robust solution for SST prediction compared with existing data-driven approaches.

It should be noted that the current quantitative comparison is reported on one representative 240 min test segment. While this setting is useful for controlled benchmarking, broader robustness assessment across longer periods and explicitly stratified extreme operating scenarios (e.g., deep peak-shaving intervals) remains necessary. Expanding the evaluation to multi-period and condition-stratified test sets, together with cross-model runtime/resource profiling, will be an important next step to further strengthen industrial credibility.

#### 5.6.2. Ablation Study

To evaluate the effectiveness of the proposed method, an ablation study is conducted to quantify the contributions of two key components—the Local Temporal Convolution (LTC) module and the physics-guided loss—to overall prediction performance. The corresponding results are presented in Table 8.

Specifically, the effects of each component are analyzed incrementally. The introduction of the LTC module leads to a noticeable improvement in prediction accuracy, as evidenced by reduced MSE and MAE, along with an increased $R^{2}$. This indicates that the LTC module effectively enhances the model’s ability to capture local temporal patterns and short-term dependencies in the time series. Furthermore, incorporating the physics-guided loss results in additional performance gains, suggesting that embedding physical constraints helps improve model generalization and ensures more physically consistent predictions.

Compared with the baseline iTransformer, the proposed iTransformer-SST achieves a 13.5% reduction in MSE, demonstrating the effectiveness of the overall design. These results are consistent with the analyses presented in the previous sections, confirming that the proposed components contribute to improved modeling of complex multivariate relationships and enhanced prediction performance.

## 6. Conclusions and Future Work

This study presents iTransformer-SST for superheated steam temperature prediction in coal-fired power plants. The core contributions are threefold: (i) a task-oriented coupling of variable-wise global dependency modeling (iTransformer) with local disturbance extraction (LTC); (ii) a time-lag-aware physics-guided objective that embeds monotonicity and smoothness priors derived from thermal-process characteristics; and (iii) a deployability-oriented evaluation perspective that links forecasting performance to edge-side timing constraints.

Several limitations should also be acknowledged. First, the current evaluation is based on data from one 1000 MW unit, so generalization to unseen units and operating regimes has not yet been fully verified. In particular, we have not conducted a dedicated sensitivity study across different data sources (e.g., plants/regions) and unit capacities (e.g., subcritical, supercritical, and ultra-supercritical scales). Second, performance under explicitly stratified extreme conditions (e.g., deep peak-shaving intervals and abrupt disturbance transitions) still requires broader multi-period testing. Third, because industrial data quality can vary with sensor drift, missing values, and communication noise, robustness under severe data-quality degradation remains an open issue.

Future work will therefore focus on three concrete implementation pathways:

1. Cross-source and cross-capacity generalization validation: build a multi-unit, multi-capacity, multi-period, and multi-source benchmark, and adopt transfer/continual adaptation protocols to evaluate and improve out-of-domain performance.
2. Condition-stratified robustness evaluation: construct test subsets by load range and operating events (including deep peak shaving), and report scenario-level accuracy, stability, and failure cases.
3. Data-quality-aware deployment enhancement: introduce sensor-quality monitoring, uncertainty-aware inference, and missing/noisy-data augmentation to improve reliability under real industrial disturbances; then integrate the predictor with MPC in closed-loop pilot tests.

Overall, iTransformer-SST provides a practically meaningful step toward physics-guided and deployment-oriented SST forecasting. The above limitations and roadmap define the next stage toward robust large-scale industrial application.

## Figures and Tables

**Table 1:** Feature numbers and corresponding names.

Number	Feature
$f_{1}$	Inlet temperature of A-side tertiary attemperator
$f_{2}$	Outlet temperature of A-side tertiary attemperator
$f_{3}$	Inlet temperature of A-side secondary attemperator
$f_{4}$	Outlet temperature of A-side secondary attemperator
$f_{5}$	Total fuel quantity after BTU correction
$f_{6}$	Actual total coal quantity
$f_{7}$	Generator power
$f_{8}$	Total air flow
$f_{9}$	Feedwater flow
$f_{10}$	Air preheater inlet oxygen content

**Table 2:** Correlation analysis results of features for the A-side subsystem.

Number	Max Corr. Coeff.	Pearson	Spearman	Lag Time (min)
$f_{1}$	0.7734	0.6982	0.6242	1.16
$f_{2}$	0.6681	0.5122	0.4047	2.00
$f_{3}$	0.4424	0.3909	0.2737	0.92
$f_{4}$	0.4294	0.3125	0.2205	2.92
$f_{5}$	0.3978	0.2627	0.1148	4.60
$f_{6}$	0.3829	0.2544	0.1206	4.60
$f_{7}$	0.3732	0.2913	0.1622	2.52
$f_{8}$	0.3619	0.2754	0.1463	3.16
$f_{9}$	0.3460	0.2643	0.1260	3.04
$f_{10}$	−0.4492	−0.3824	−0.2111	0.92

**Table 3:** Correlation analysis results of features for the B-side subsystem.

Number	Max Corr. Coeff.	Pearson	Spearman	Lag Time (min)
$f_{1}$	0.7305	0.6786	0.6041	0.92
$f_{2}$	0.6079	0.4919	0.3536	1.88
$f_{3}$	0.4417	0.3810	0.2525	1.28
$f_{4}$	0.4003	0.3013	0.1805	2.76
$f_{5}$	0.3785	0.2520	0.1036	4.48
$f_{6}$	0.3637	0.2436	0.1104	4.48
$f_{7}$	0.3577	0.2748	0.1412	2.04
$f_{8}$	0.3406	0.2596	0.1213	2.88
$f_{9}$	0.3272	0.2484	0.1010	2.64
$f_{10}$	−0.4396	−0.3719	−0.2001	0.88

**Table 4:** Effect of Training Epochs.

Epoch	MSE	MAE	$R^{2}$
5	0.0966	0.2383	0.9642
10	0.0921	0.2254	0.9595
20	0.0914	0.2311	0.9650
30	0.1233	0.2583	0.9595

**Table 5:** Effect of Learning Rate.

Learning Rate	MSE	MAE	$R^{2}$
0.00001	0.1243	0.2684	0.9554
0.00005	0.0995	0.2356	0.9602
0.0001	0.0914	0.2311	0.9650
0.0005	0.1277	0.2726	0.9595
0.001	0.1028	0.2408	0.9603

**Table 6:** Model Hyperparameters.

Hyperparameter	Value
Number of Attention Heads (n_heads)	8
Model Dimension (d_model)	512
Feed-Forward Dimension (d_ff)	2048
Number of Encoder Layers (e_layers)	2
LTC Kernel Size	3
Number of LTC Branches	2
Dilation Rates	(1, 2)
LTC Pointwise Fusion	1×1 conv (1024→512)
$\lambda_{1}$	0.2
$\lambda_{2}$	0.1
Batch Size	128
Learning Rate	0.0001
Dropout Rate	0.1
Epochs	20

**Table 7:** Model performance comparison.

Model	MSE	MAE	$R^{2}$
LSTM	0.6301	0.5895	0.7504
Informer	0.1443	0.2914	0.9461
iTransformer	0.1025	0.2386	0.9595
iTransformer-SST	0.0887	0.2312	0.9650

**Table 8:** Ablation study of iTransformer variants.

Model	MSE	MAE	$R^{2}$
iTransformer	0.1025	0.2386	0.9595
iTransformer + LTC	0.0899	0.2329	0.9645
iTransformer-SST	0.0887	0.2312	0.9650

## Data Availability

The data presented in this study are available on request from the corresponding author. The data are not publicly available due to privacy and confidentiality concerns.
